# The Serological Response in Cattle following Administration of a Heterologous Sheep Pox Virus Strain Vaccine for Protection from Lumpy Skin Disease; Current Situation in Armenia

**DOI:** 10.3390/vetsci10020102

**Published:** 2023-01-31

**Authors:** Varduhi Hakobyan, Khachik Sargsyan, Satenik Kharatyan, Hasmik Elbakyan, Vazgen Sargsyan, Tigran Markosyan, Tigranuhi Vardanyan, Manvel Badalyan, Jenna E. Achenbach

**Affiliations:** 1 Scientific Center for Risk Assessment and Analysis in Food Safety Area, 107/2 Masis Highway, Yerevan 0071, Armenia; 2 General Biology Department, Armenian National Agrarian University, 74 Teryan Street, Yerevan 0009, Armenia; 3 Battelle Memorial Institute, 1001 Research Park Boulevard, Town Center Two, Suite 400, Charlottesville, VA 22911, USA

**Keywords:** cattle, lumpy skin disease, vaccination, heterologous vaccine, ticks, control

## Abstract

**Simple Summary:**

Lumpy skin disease (LSD) is an economically important viral disease of cattle caused by the lumpy skin disease virus (LSDV) of the Poxviridae family. It was first reported in Armenia in 2015 when the disease was rapidly identified with no further spread from the initial outbreak. This was followed by a vaccination campaign to help keep the disease from returning. Vaccination can be a powerful tool for preventing the spread of transboundary diseases, but there is currently no single vaccine that protects cattle from all strains of LSD, with many vaccines providing various results. We sought to assess the quality of vaccination currently used in Armenia by evaluating the antibody response of a dry culture sheep pox virus-based vaccine against LSD in cattle. Cattle were able to produce protective antibodies >80%, which should protect against lumpy skin disease, based on previous observations, but suggests more work is needed. We have also observed no adverse side-effects in cattle following vaccination. In an area that is not suffering from continuous outbreaks, we suggest this vaccine could be a safe alternative for protection from LSD in cattle.

**Abstract:**

Lumpy skin disease (LSD) is a highly infectious viral disease of cattle caused by LSD virus (LSDV), which was first reported in Armenia in late 2015. It was identified in pasture-raised cattle near the border with Iran. Currently, vaccination plays a key role in preventing further incursion of disease in high-risk areas. The purpose of this work was to assess the quality of vaccination currently used in Armenia by determining the immune response of the heterologous dry culture sheep pox virus-based vaccine against LSD in cattle. Seroprevalence and seroconversion testing was carried out using an ELISA to detect specific antibodies against LSD before and 30 days after vaccination in three adjacent regions of Armenia (Ararat, Armavir, Gegharkunik). *Ixodes* ticks were also examined for the presence of LSDV via real-time PCR. We found that the heterologous vaccine used in Armenia creates a high level of population immunity of 86.09% (83.83–87.97%) and no adverse side effects were observed in cattle. Of the 6 types of *Ixodes* ticks identified and tested, we found no evidence of LSDV circulating in these vectors. These results suggest that regular serological monitoring via ELISA and heterologous vaccination should continue in areas of Armenia at high risk for incursion of LSD to reduce the spread of this highly infectious transboundary disease.

## 1. Introduction

Lumpy skin disease (LSD) is a transboundary especially dangerous pathogen (EDP) affecting cattle with low mortality that can lead to significant production losses. Due to the potential risk of a rapid spread, the World Organization for Animal Health (WOAH) lists LSD as a notifiable disease [1,2,3,4].In cattle, LSD causes fever, watery eyes, nasal discharge, excessive saliva, reduced appetite, and lumps on the skin that can become ulcers that could be a source of infectious virus spread. The disease can also lead to reduced milk production, loss of physical condition, and rejection of hides for commercial use due to scarring.

The causative agent of LSD is a virus belonging to the genus Capripoxvirus of the *Poxviridae* family. The genus Capripoxvirus includes sheep pox virus, goat pox virus, and LSD, with high antigenic relatedness among all three. Cattle and Asian buffaloes are susceptible to LSD regardless of breed, sex, age, or lactating status. Cattle that have recovered from LSD provide protection to newborn calves through colostrum up to 6 months of age. Additionally, there have been isolated reports of LSD morbidity in sheep and goats [5].

The established route of transmission of LSD virus is by biting vectors of mosquitos of genus *Culex* and *Aedes*, biting flies of genus *Stomoxys* and *Biomyia*, and *Ixodes* ticks, all of which are considered as mechanical carriers of the infectious agent [6,7,8,9,10,11,12].

The keys to successful control and eradication of LSD in cattle is early detection of disease foci with prompt laboratory confirmation, slaughter during primary outbreaks, strict control of movement of animals, quarantine, disinfection, increasing the level of biosecurity of farms, and vaccination. To prevent the spread of LSD outside of areas at greatest risk and to eliminate future outbreaks, susceptible animals within the epizootic foci are vaccinated. Currently, there are two types of live attenuated vaccines against LSDV on the world market: homologous vaccines, consisting of LSDV strains, and heterologous vaccines, based on sheep pox virus or goat pox virus strains [13]. For the control of LSD, the most commonly used vaccines in cattle are live attenuated vaccines based on sheep pox virus (Kenyan sheep pox (KS1 O-180), Romanian sheep pox, and Yugoslavian RM 65 sheep pox vaccines), goat pox virus (Gorgan goat pox vaccine), and LSDV (Neethling strain vaccine) [13,14,15,16].

Expert opinions on the efficacy and safety of these vaccines vary. There has been success with homologous vaccines, but there is a potential for post-vaccination complications in vaccinated animals. Others consider heterologous vaccines to be safer, although studies have shown them to be less effective against LSD than homologous vaccines [17].Live attenuated LSD vaccines provide good herd protection if ≥80% of the animals are vaccinated, which can be maintained through annual vaccination [4].

Sheep pox and goat pox viruses are genetically similar to LSDV and vaccination with goat pox or sheep pox viruses induces cross-immunity. Some scientists [18,19] believe that the use of homologous vaccines in vaccinated animals may result in lesions on the skin containing high titers of the virus that can become a source of its spread through vectors. For this reason, they are not recommended for use in LSD-free countries due to potential safety concerns. Sheep pox and goat pox vaccines have been used in cattle to protect from LSD with differing efficacy [13,14,15,20,21,22,23,24,25,26], but side-effects are rare following the sheep pox vaccine compared to attenuated LSD vaccines [13] making their use in countries where the disease is absent more appealing for controlling disease.

Data is available on the humoral immunity in animals following vaccination with LSD vaccines. Antibodies are commonly measured via ELISA and serum or virus neutralization tests, with antibodies being detected as early as 10–15 days following vaccination, reaching a peak by 30 days, after which the titers begin to drop [13]. Additionally, it has been shown that LSD-specific antibody titers could be detected as late as 46–47 weeks [27], but there is no direct correlation between antibody titers and protection from infection. Similarly, studies of the antibody response in calves following vaccination with the Gorgon goat pox vaccine showed detectable antibody titers at 21 days post vaccination;however, there was no detectable antibody response in calves to the two LSD vaccine strains at the same time point, but post challenge, antibody titers were detectable earlier in calves vaccinated with LSDV strains [14]. Differences in delayed type hypersensitivity were also evaluated in this study and the response was highest in calves subcutaneously vaccinated with the goat pox strain suggesting higher immunogenicity. There could be differences in the vaccinations due to the development of the vaccine itself and the genetic relatedness to the challenge virus but there are still many questions to be answered to develop the best vaccine for control of LSD.

Throughout Africa, the Middle East, Asia, and the Pacific regions, millions of cattle are immunized yearly, yet outbreaks of LSD continue to occur. This can be explained by the fact that certain aspects of the epidemiology of LSD are not fully elucidated, and various elements such as the selection of the best vaccine and the role of ticks and mechanical vectors in transmission and animal behavior are not taken into account when planning and conducting preventive measures that influence the spread of disease.

Prior to 2015, LSD had never been reported in Armenia. The first clinical cases of LSD were reported on 8 December 2015 in the southern part of Armenia in the Syunik region (near the border with Iran) among cattle that were kept on pastures. Samples taken from sick animals were positive for LSDV when tested via real-time PCR, and a confirmatory diagnosis was made at the Federal Center for Animal Health, FGB ARRIAH (Vladimir, Russian Federation). To control the outbreak and prevent the further spread of disease, vaccination with a heterologous sheep pox vaccine against sheep pox and lumpy skin disease sheep pox live vaccine was implemented.

During 2016, 344,312 cattle were vaccinated, which accounted for 49% of the total population of cattle in Armenia. In the Syunik region where the outbreak occurred, 95,233 cattle were vaccinated, which is 27.6% of all vaccinated cattle across the 10 regions of Armenia. In 2016, approximately 50% of the total cattle population were in high-risk zones and were vaccinated in parallel with additional control measures. The additional control measures included a strict quarantine in the infected communities, control of vectors, disinfection efforts, controlled movement of animals inside the country, and surveillance screening both outside and within the containment or protection zone. Following the initial outbreak, no further LSD outbreaks occurred.

**Figure 1:**
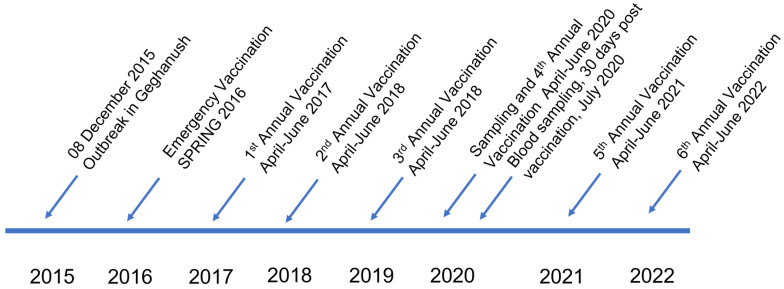
Outbreak on 8 December 2015 in the Syunik region. This was followed by an emergency vaccination campaign cattle during the spring of 2016 with a sheep pox virus vaccine strain. Vaccination covered ~50% of all cattle at risk. Due to the epizootic situation in neighboring countries with LSD, scheduled preventive vaccinations are carried out annually in Armenia during the 2nd quarter of each year. Blood sampling in cattle was performed in 2020 for this study.

In 2017, 143,206 cattle were vaccinated with 61,961 in the Syunik region. The total number of vaccinated cattle in 2017 was expected to be less as there were no new registered cases of LSD in other regions of the country so targeted vaccination occurred mainly in the Syunik region with additional susceptible cattle vaccinated in other high-risk regions based on shared borders with countries such as Turkey, Georgia, Azerbaijan, and Iran that were having outbreaks at the time or continue to have outbreaks (https://wahis.woah.org/#/dashboards/country-or-disease-dashboard; accessed on 23 January 2023). Susceptible cattle continued to be vaccinated in high-risk areas through 2022 (Figure 1).

To improve our understanding of vaccine requirements and evaluate our current vaccination regime, we sought to analyze the level of antibody response in cattle to LSD following field vaccination with a heterologous sheep pox virus vaccine strain. We performed a serological study before and after vaccination in young unvaccinated cattle in three regions which are at risk for incursion of LSD and where we currently perform vaccination of susceptible animals against LSDV.

## 2. Materials and Methods

### 2.1. Study Area

We conducted seromonitoring studies to detect LSDV antibodies in three regions of Armenia (Ararat, Armavir, and Gegharkunik).All three are within the high-risk zones for introduction and spread of this disease. The high-risk zones border Turkey and Azerbaijan and are swamp environments favorable for insects and mosquitoes that can transmit LSD. This area also includes animals that have been vaccinated against LSD since the first outbreak in Armenia in 2016.

### 2.2. Sample Size

The sample size calculation was determined applying a 95% confidence level, using the following tools: http://www.winepi.net and http://www.epitools.ausvet.com.au; accessed on 23 January 2023. The population size was determined using statistical data from the veterinary services using an expected prevalence of 2% with a 5% accepted error. Within the three regions, we utilized the targeted randomized cluster method to select the samples. Simple random sampling was applied at the first stage giving every village the same chance of being selected and stratified at the region level. The second stage used random sampling of the animals in villages. The number of samples used in the study washigher than the calculated numbers.

### 2.3. Sample Collection

This trial was carried out under the agreement of the Scientific Council of the Scientific Center for Risk Assessment and Analysis in Food Safety Area, with the protocol approved in February 2021.

Sampling was conducted in 60 animals prior to vaccination in unvaccinated cattle up to 6 months of age, excluding maternal immunity, and again 30 days after vaccination to evaluate the presence of antibodies. After vaccination, we monitored the animals for adverse reactions for 3–4 days. We also measured body temperature and evaluated the presence of swelling at the injection site.

**Figure 2:**
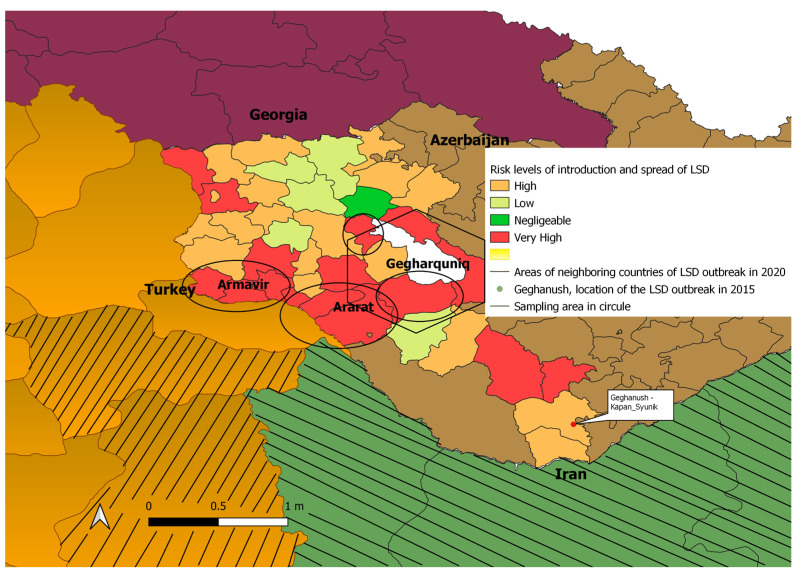
Risk levels of LSDV incursion and locations of sampling and vaccination in Armenia in 2020 with neighboring countries showing outbreaks at that time.

We collected a total of 60 blood samples from unvaccinated cattle prior to vaccination and 798 blood samples from cattle 30 days after vaccination. Samples were collected in 14 communities randomly selected from each region with 19 animals sampled from each community resulting in 266 samples from each of the 3 regions (Figure 2). The sample size calculation was determined by applying a 95% confidence level, using the following tools: http://www.winepi.net and http://www.epitools.ausvet.com.au; accessed on 23 January 2023. The program accounted for the number of susceptible animals, possible risks for introduction and the spread of disease using an expected prevalence of LSD of 2%.Animals in the village were selected randomly or, if logistically not possible, via methods that ensured a representative selection and included more than 3 animals per owner.

Blood was collected from cattle via the jugular vein into vacutainers with a red cap to harvest serum and delivered to the laboratory in cold boxes within 24 h. In the laboratory, the serum was separated and aliquoted into Eppendorf tubes and labeled for further analysis.

Given that Ixodes ticks play a major role in transmission of LSD we also collected a total of 250 Ixodes ticks evenly across the 3 regions (Ararat, Armavir, Gegharkunik). Ticks were collected in the spring–summer period from the pastures and from animals, as well as animals from which blood samples were collected.

### 2.4. Vaccination

Susceptible cattle were vaccinated with the 10× Sheep Pox Cultural Dry™ vaccine produced by the Federal Centre for Animal Health (ARRIAH) in 2016–2022. In 2020, vaccination was carried out in May–June with a total of 36,418 cattle vaccinated in the Ararat region, 49,174 in Armavir, and 74,710 cattle in the Gegharkunik region, which are reported in this study. Vaccination continued throughout 2021 and 2022. Vaccination plans and implementation for 2020 can be seen in Table 1.

### 2.5. Detection of Antibodies in Cattle

Serum samples were tested for the presence of antibodies to LSDV using ID Screen Capripox Double Antigen Multi-species ELISA (IDVet, Grabels, France, https://www.innovative-diagnostics.com/produit/id-screen-capripox-double-antigen-multi-species/; accessed on 23 January 2023) [28] recommended by the OIE Guidelines for Diagnostic Tests and Vaccines for Terrestrial Animals and performed according to the manufacturer instructions. This ELISA is designed to detect antibodies against Capripoxviruses including LSDV, sheep pox virus, and goat pox virus. Samples are read at 450nm and considered positive when the S/P% of the sample ≥30%. The S/P% is calculated according to: (OD of the sample/OD of the positive control) × 100.

### 2.6. Detection of LSDV in Ticks

Following the morphological identification of the ticks, tick homogenates were subjected to PCR to determine the presence of LSDV. To obtain a homogenate suspension, ticks were ground using a sterile porcelain mortar and pestle, then combined with nuclease-free water to obtain a 10% suspension. Total DNA was extracted from the tick homogenate using aDNeasy Blood and Tissue kit (Qiagen, Valencia, CA, USA), according to the manufacturer instructions (https://www.qiagen.com/us/products/discovery-and-translational-research/dna-rna-purification/dna-purification/genomic-dna/dneasy-blood-and-tissue-kit; accessed on 23 January 2023).

The SD real-time PCR screening test from the ARRIAH (Vladimir, Russia) was utilized to detect field isolates of LSDV. Real-time PCR was performed with a TProfessional basic thermocycler (Biometra Ltd., Göttingen, Germany) according to the manufacturer instructions. Briefly, the reaction was carried out as follows: activation at 95 °C for 5 min (1 cycle); amplification 40 cycles of 95 °C for 15 s, 60 °C for 1 min. We included both a negative and positive control during each run. The presence of LSDV DNA in the sample was confirmed positive if the value of Ct was 35 or higher and negative if the Ct value was below 35.

## 3. Results

### 3.1. Serological Results

To assess the effectiveness of vaccination and determine the immune background of vaccinated cattle against LSD in 3 regions of Armenia, blood serum samples were analyzed using ELISA before and after vaccination to determine seroprevalence and seroconversion. Seroprevalence studies that were performed on collected samples prior to vaccination showed no detectable LSDV antibodies via ELISA in the 60 cattle tested (Table 2).

After vaccination serum samples were taken from the same farms and analyzed via ELISA to assess seroconversion. The individual results of the 60 cattle that had pre-vaccination blood titer tested are presented in Table 2. The overall results of the studies conducted across the 3 regions on the 30th day after vaccinations were analyzed by region and are shown in Table 3.

Thirty days after vaccination, 687 out of 798 examined animals were found to have antibodies to LSDV, and based on seroconversion values, the level of immune animals averaged 86.09% (83.83–87.97%).

### 3.2. Vaccination Safety

On the day of vaccination and throughout the study period, all investigated cattle were healthy. Following vaccination, we observed no swelling at the injection site or any other side effects of vaccination. Overall, we observed no increase in body temperature or deviation from the physiological norm in all cattle.

### 3.3. Tick Species and LSDV Identification

Considering the role of ticks in the transmission of LSD, we also analyzed ticks for species identification and to determine the presence of the LSDV. In total, 250 *Ixodes* ticks were collected from the pastures and from animals that belong to the following species: *Dermacentor reticulates* (95), *D. marginatus* (51), *Boophilusannulatus* spp. (48), *Rhipicephalus bursa* (23), *Hyalomma asiaticum caucasicum* (19), and *H.anatolicum* (14). PCR testing of ticks determined that LSDV was not detected in any of the tick samples.

## 4. Discussion

Prior to 2015, LSD had not been registered in Armenia. The first clinical cases of LSD were observed in the southern part of Armenia (near the border with Iran) at the end of 2015 in cattle which were kept on pastures [29]. Samples collected from sick animals were positive for LSDV when tested via real-time PCR with a confirmatory diagnosis established via the ARRIAH [30].

Following LSDV detection and our initial investigations into the LSD outbreak in 2015–2016, Armenia began a vaccination campaign in susceptible cattle in high-risk areas and performed passive surveys. Vaccination is the most effective way to prevent the spread of the infection in endemic and newly affected regions. Indeed, studies have shown that several countries in southeastern Europe and Israel have successfully prevented LSDV incursions by vaccinating the susceptible population [31]. Yet, in the event of an outbreak, selection of the best vaccine presents a significant challenge for veterinary authorities and farmers. Decision makers need sound scientific information to support their decisions and subsequent actions. The currently available vaccines differ in their quality and efficacy and various differences have been reviewed [13]. This includes the inadequate protection afforded to cattle with the use of the Yugoslavian strain (RM65) or Romanian strain of sheep pox in cattle in Egypt, Morocco, and Israel [21,22,23] and the clarification of the Kenyan sheep and goat pox vaccine virus (KSGP) O-240 strain that was genetically determined to be LSDV but was noted as not sufficient to protect cattle from LSDV due to the low level of attenuation [15]. Additionally, there have been reports of adverse reactions in cattle when using other sheep pox vaccines (POXVAX-10X and PENPOX-3X, both produced in Turkey and used in Georgia and Turkey respectively) [32]; however, we have not observed any adverse reactions with the ARRIAH strain nor development of lesions, which has been noted following administration of homologous LSD strains [33], although it should be noted that a homologous LSDV vaccine was successfully used in the Balkans to eradicate the disease [34]. There has been preliminary data for both sheep and cattle with the ARRIAH dry culture sheep pox virus vaccine [35,36,37,38]. These have been limited in the numbers of animals tested, the methods of the testing, and the lack of a controlled challenge study to fully assess the vaccine and its ability to protect cattle from LSDV. The most similar conditions for evaluating the vaccine come from Ragimkhanovich et al., 2017, from their patent application for the ARRIAH sheep pox virus vaccine [38]. They evaluated the vaccine in cattle in the field in an area with risk for outbreaks of LSDV. They utilized the vaccine at a 10×dose such as performed in Armenia and vaccinated 2438 heads of cattle via subcutaneous injection. Following vaccination, an outbreak occurred and 105 (4.3%) of the vaccinated cattle developed LSD with 2388 (95.7%) not developing disease [38]. Unfortunately, they did not show any seroconversion titers of cattle following vaccination in this report, which limits comparison and reduces any further assessment. They also do not mention the field strain of LSDV that caused the outbreak, nor did they provide any comparison to the vaccine strain. There was an additional study assessing the immune response in Hereford cattle following vaccination with the ARRIAH sheep pox virus vaccine [35,36]. They reported overall development of antibodies in three groups of cattle: (1) vaccination alone, (2) vaccination plus interferon-B, and (3) vaccination plus Asidivit, the latter two being used to boost the immune response to vaccination with this vaccine in cattle. Their vaccination alone group showed 40% of the animals developed positive antibodies with the two treatment groups developing positive antibodies in 60% and 80%, respectively [35]. There were also caveats with this publication as they do not mention the dose of sheep pox virus vaccine given to the cattle and the numbers of cattle tested were very low. They do mention that they did not detect any live virus following vaccination nor did they observe any adverse reactions to the vaccine. Currently, the veterinary services of Armenia are successfully using the heterologous Sheep Pox Cultural Dry™ vaccine produced by the ARRIAH to curb the spread of LSD [32].

Our study has limitations. There have been no further outbreaks of LSD in Armenia since the identification in 2015 so the efficacy of the vaccine cannot be fully elucidated with absence of disease. More studies are needed including challenge experiments to verify the efficacy of this specific vaccine against LSDV in cattle.

Even though no LSD outbreaks have been reported in Armenia following the implementation of a comprehensive vaccination campaign, the status of immunity against LSDV was unknown. Here, we sought to investigate the humoral immune response of vaccinated cattle before and after vaccination with a heterologous sheep pox vaccine. Humoral immune response, assessed through seroconversion, can provide significant information about the success of vaccination. Previous studies have shown that the commercially available double antigen ELISA is as specific as virus neutralization assays and eliminates the need to use live virus providing an accurate but safer laboratory assay for detection of LSDV-specific antibodies in field serological monitoring studies [27].

Prior to vaccination, none of the tested cattle had detectible antibodies using ELISA. Following 30 days after vaccination, 86.09% (83.83–87.97%) of the studied animals seroconverted with detectable antibodies to LSDV. This has been shown in previous publications with the development of lower neutralizing antibody titers following vaccination with a sheep pox virus vaccine in cattle [22,35]. This could be due to the concentration or titer of the vaccine and it could be related to the vaccinated animal having a concomitant disease, maternal antibodies, and cold chain maintenance. With many vaccines it is true that animals may have a low antibody response but are still protected from disease. Indeed, there are examples of animals not seroconverting following vaccination but still being protected from disease [27,39]. Additionally, work with foot-and-mouth disease vaccines have shown difficulty in relating a neutralizing antibody or serum antibody response that directly correlates with protection. Previous work with attenuated LSDV vaccines has shown that protection is afforded when vaccination coverage reached 80% [3], and it is expected this should be higher when dealing with a heterologous vaccine. This suggests that after vaccination with this heterologous sheep pox virus vaccine against LSD the level of antibodies produced is >80%, which maybe sufficientfor protection against LSD infection and, contrary to homologous LSDV vaccines, has been shown to not cause side effects [17,18,19]. More experimental research is needed to fully elucidate this protection as other strains of sheep pox virus vaccines have shown reduced efficacy, which suggests definite variation in strains and titers of vaccine used [14,20,21,23,36].

Of the six species of *Ixodes* ticks tested, we found no evidence of LSDV circulating in these vectors. This can be explained by the fact that the first case of LSD was registered in December 2015and during this period ticks were not active in Armenia.The disease was quickly eradicated, and as a result, ticks did not have the opportunity to be infected.

## 5. Conclusions

Based on our results that show a high antibody response to LSDV following administration of a heterologous vaccine, we conclude that the regular annual vaccination of cattle, especially of animals located on the border with Turkey and Iran where LSD is currently registered [40], should provide protection to all cattle from new infections. Additionally, cattle imported from Europe and Russia are placed in strict quarantine for 21–28 days and undergo specific testing protocols to restrict the direct importation of transboundary diseases. These implemented quarantine and vaccination measures have kept Armenia from further outbreaks of LSD and should be continued for long-term protection.

## Figures and Tables

**Table 1:** Regions covered in this study, the planned number for vaccination, and the numbers of fully vaccinated animals in 2020. Vaccination occurred April–June 2020.

Region	Area	Number of Cattle	Plan Vaccination	Vaccinated Animals	Vaccination Coverage
Ararat	Ararat	15,464	13,918	13,996	90.5
	Artashat	13,444	12,100	12,211	90.8
	Masis	11,272	10,145	10,211	90.6
Armavir	Ejmiatsin	13,314	12,427	12,457	93.6
	Armavir	29,543	26,589	26,621	90.1
	Baghramyan	11,217	10,095	10,096	90.0
Gegharquniq	Sevan	12,613	12,613	12,612	99.99
	Martuni	29,585	29,585	29,617	100.1
	Vardenis	23,114	14,215	14,271	61.7
	Gavar	17,610	10,830	10,822	61.5
	Chambarak	12,002	7381	7388	61.6

**Table 2:** Individual seroconversion results of 60 cattle with pre- and 30 days post-vaccination titers.

Region	Cattle	Before Vaccination S/P%	30 Days after Vaccination S/P%
Ararat	Cattle 1/1	0.7	49.9
	Cattle 1/2	17.5	85.2
	Cattle 1/3	3.7	56.7
	Cattle 1/4	0.1	60.4
	Cattle 1/5	6.7	82.0
	Cattle 1/6	1.9	45.9
	Cattle 1/7	18.2	91.4
	Cattle 1/8	19.6	89.4
	Cattle 1/9	0.4	45.9
	Cattle 1/10	6.7	48.3
	Cattle 1/11	1.9	59.5
	Cattle 1/12	2.5	36.1
	Cattle 1/13	5.5	52.0
	Cattle 1/14	−1.4	52.4
	Cattle 1/15	−0.2	89.4
	Cattle 1/16	3.9	82.7
	Cattle 1/17	−2.4	45.1
	Cattle 1/18	0.4	49.9
	Cattle 1/19	0.9	82.5
	Cattle 1/20	9.9	91.4
Armavir	Cattle 2/1	−1.7	52.0
	Cattle 2/2	9.8	59.2
	Cattle 2/3	−0.1	45.9
	Cattle 2/4	8.0	61.2
	Cattle 2/5	0.4	56.5
	Cattle 2/6	−2.1	44.1
	Cattle 2/7	−1.6	38.6
	Cattle 2/8	8.0	63.6
	Cattle 2/9	9.3	51.3
	Cattle 2/10	6.3	62.2
	Cattle 2/11	11.3	57.2
	Cattle 2/12	1.7	35.1
	Cattle 2/13	14.6	78.8
	Cattle 2/14	11.4	76.1
	Cattle 2/15	1.9	41.6
	Cattle 2/16	2.0	46.9
	Cattle 2/17	−2.4	35.1
	Cattle 2/18	−0.5	49.8
	Cattle 2/19	3.9	68.7
	Cattle 2/20	5.5	74.9
Gegharqunik	Cattle 3/1	11.1	65.4
	Cattle 3/2	9.4	66.3
	Cattle 3/3	7.9	58.4
	Cattle 3/4	3.4	46.9
	Cattle 3/5	3.2	41.6
	Cattle 3/6	0.6	37.5
	Cattle 3/7	2.1	35.1
	Cattle 3/8	2.4	52.2
	Cattle 3/9	0.9	42.1
	Cattle 3/10	0.4	43.1
	Cattle 3/11	5.6	76.0
	Cattle 3/12	0.6	37.1
	Cattle 3/13	3.6	52.1
	Cattle 3/14	12.1	59.6
	Cattle 3/15	18.9	93.7
	Cattle 3/16	3.4	59.1
	Cattle 3/17	1.6	41.6
	Cattle 3/18	6.2	73.9
	Cattle 3/19	8.9	74.9
	Cattle 3/20	11.8	85.3

**Table 3:** ELISA antibody titers in cattle 30 days after vaccination with sheep pox vaccine against LSD in each of 3 regions.

Regions	Number of Examined Samples	30 Days after Vaccination			
		Seronegative		Seropositive	
		n	%	n	%
Gegharkunik	266	43	16.17	223	83.83
Armavir	266	36	13.53	230	86.47
Ararat	266	32	12.03	234	87.97
TOTAL	798	111	13.91 *	687	86.09 *

n = number of samples; * = mean percent of negative or positive samples across the 3 regions.

## Data Availability

The datasets analyzed during the current study are available from the corresponding author upon reasonable request.
